# Reasoning, cognitive control, and moral intuition

**DOI:** 10.3389/fnint.2012.00114

**Published:** 2012-12-18

**Authors:** Richard Patterson, Jared Rothstein, Aron K. Barbey

**Affiliations:** ^1^Philosophy Department, Emory UniversityAtlanta, GA, USA; ^2^Department of Behavioral and Social Sciences, Daytona State CollegeDaytona Beach, FL, USA; ^3^Decision Neuroscience Laboratory, University of Illinois at Urbana-ChampaignChampaign, IL, USA; ^4^Beckman Institute for Advanced Science and Technology, University of Illinois at Urbana-ChampaignChampaign, IL, USA; ^5^Department of Internal Medicine, University of Illinois at Urbana-ChampaignChampaign, IL, USA; ^6^Department of Psychology, University of Illinois at Urbana-ChampaignChampaign, IL, USA; ^7^Department of Speech and Hearing Science, University of Illinois at Urbana-ChampaignChampaign, IL, USA; ^8^Neuroscience Program, University of Illinois at Urbana-ChampaignChampaign, IL, USA

**Keywords:** intuition, reasoning, decision neuroscience, affect, neurosciences

## Abstract

Recent Social Intuitionist work suggests that moral judgments are intuitive (not based on conscious deliberation or any significant chain of inference), and that the reasons we produce to explain or justify our judgments and actions are for the most part *post hoc* rationalizations rather than the actual source of those judgments. This is consistent with work on judgment and explanation in other domains, and it correctly challenges one-sidedly rationalistic accounts. We suggest that in fact reasoning has a great deal of influence on moral judgments and on intuitive judgments in general. This influence is not apparent from study of judgments simply in their immediate context, but it is crucial for the question of how cognition can help us avoid deleterious effects and enhance potentially beneficial effects of affect on judgment, action, and cognition itself. We begin with established work on several reactive strategies for cognitive control of affect (e.g., suppression, reappraisal), then give special attention to more complex sorts of conflict (“extended deliberation”) involving multiple interacting factors, both affective and reflective. These situations are especially difficult to study in a controlled way, but we propose some possible experimental approaches. We then review proactive strategies for control, including avoidance of temptation and mindfulness meditation (Froeliger et al., 2012, this issue). We give special attention to the role of slow or “cool” cognitive processes (e.g., deliberation, planning, and executive control) in the inculcation of long-term dispositions, traits, intuitions, skills, or habits. The latter are critical because they in turn give rise to a great many of our fast, intuitive judgments. The reasoning processes involved here are distinct from *post hoc* rationalizations and have a very real impact on countless intuitive judgments in concrete situations. This calls for a substantial enlargement of research on cognitive control, drawing on work in developmental psychology, automatization, educational theory, and other fields.

It seems obvious that on occasion people make impulsive judgments or decisions and live to regret it. What to do about this is less obvious, but traditionally common sense and philosophy have agreed that reason offers some hope: “think it over before you decide,” “look before you leap.” Most people are inclined to think that reasoning, if given half a chance, can help us recognize some impulses or intuitions as foolish and others as sound. We can then give due weight to sound intuitions and use various means to counter the force of foolish ones. But does reasoning actually have this much influence? Recent work by “Social Intuitionists” on decision-making and moral judgment in particular (Haidt, 2001; Greene, 2007) suggests that almost everybody, almost all the time, exercises moral judgment via quick, intuitive (non-deliberative), “hot” (affect laden) processes. The reasons people produce to explain or justify their moral judgments are for the most part *post hoc* rationalizations, and not the actual basis or cause of those judgments. There are exceptions, but these are scarce in everyday life and as a rule restricted to people specifically trained to think through moral judgments, conflicts or dilemmas. The same holds in many domains: people often cannot give coherent explanations of their judgments or actions at all, yet cling to those judgments nonetheless [see Mills and Keil (2004) for experimental evidence]. The real explanation for their inability to produce a coherent, defensible account is that their judgments are not based on reasoning, but are intuitive and the result of processes not available to conscious reflection. This is the basic Social Intuitionist picture of moral judgment.

Our view is that although there is much truth in this picture, there is also much left out. Above all it underestimates the causal influence of slower, controlled cognitive processes on affect and emotion on at least three fronts: first, reactive strategies for control of impulse, intuition, or emotion; second, proactive measures to avoid unwanted impulses from arising in the first place, to prepare for coping with them if they do arise, or to actively arouse affect that might in turn have enhanced effects on action or cognition; and third, proactive inculcation, (re)shaping, or elimination of the underlying bases of many of our fast, intuitive or “gut” responses. These bases include character traits, habits of perception, expertise of various sorts, dispositions to intense gut reactions such as disgust, and others. In this third area we also review interesting new work on mindfulness meditation and its influence on the downstream effects of emotional processing.

In the larger picture we see these causal roles for reasoning as part of a general theory of cognition/affect interaction that incorporates rather than contests recent work by Social Intuitionists. Our reservations about Social Intuitionism have to do not with the claim that most of our moral judgments are intuitive, but that it takes too narrow a view of the potential role of reasoning in generating or shaping those intuitive judgments, hence in influencing them for better or worse.

## Reactive regulation of affect and emotion

### Strategies for cognitive control

There are several experimentally established reactive strategies for cognitive control, including suppression, distraction, distancing (adopting a third-party view of a conflict), and reappraisal of a conflict situation, especially of the option favored by impulse or intuition (see the reviews by Ochsner et al., 2002; Kim and Hamann, 2007; Goldin et al., 2008). We focus here on reappraisal. The object of an intuitive or impulsive response—for example, my next cigarette—may initially appear to me as a source of pleasure (“Satisfying, and they are mild!”). But if I am trying to quit smoking I can consciously reappraise the cigarette as a “coffin nail,” or think of smoking as a “smelly, obnoxious habit.” Reappraisal is pervasive in advertising, political campaigns (Lakoff and Johnson, 1980) and elsewhere, and often plays a role in moral decision-making. Social Intuitionists would rightly point out that reappraisals frequently amount to rationalizations. At times these rationalizations can be quite self-serving: “If I don't take over Joe's old job (after Joe has been unjustly fired) someone else will; so I might as well do it.” In fact I may already intend to take the job. But in light of my self-serving rationalization, taking the job appears not as acquiescence or participation in an injustice, but as something that is for all practical purposes harmless. And my refusing the job now appears not as a protest against injustice but as a pointless sacrifice on my part. I may well even believe that these rationalizations explain and justify my taking the job, when in fact the real explanation is simply that I am an opportunistic cad.

Reappraisal is not limited to internally generated rationalizations, however. As Social Intuitionists point out, (re)appraisal often works by means of one person triggering a gut or intuitive response in someone else. Thus, an anti-abortionist displays a photo of a 6-months-old fetus to a freedom-of-choice advocate: “Abortion until the last trimester allows the killing of a living creature such as this.” This may stimulate a new gut reaction and a different intuitive moral judgment. But we suggest a major addition to this characterization of the new judgment as “intuitive.” For the strategy of showing such photos, or of erecting billboards graphically depicting the internal ravages of smoking, may itself have been well-researched, long-planned and executed, by epidemiologists, statisticians, social activists, publicists, legislators, and so on. The end result may well be a fast, non-deliberated, affect laden reaction on someone's part—or millions of such responses if a campaign of public education is widely successful. But these automatic, intuitive reactions are to a significant extent the causal effects of previous slow, controlled processes of thought and planning. Thus for the purpose of discovering how reasoning might influence affect, impulse, or intuitive judgment, it is crucial to go beyond the fact that a given response considered in its immediate context is fast and intuitive rather than deliberated. This holds whether or not people are aware of how their own intuitions were shaped.

Recent work on reappraisal indicates that people who use this strategy more frequently also use it more effectively (Cohen et al., 2012). This result raises further questions for investigation, including that of whether people can through practice improve their ability to use this strategy. Interestingly, Kanske (2012, this issue) finds that “temperament trait effortful control” correlates with “enhanced (task-relevant) emotion-induced facilitation of conflict processing,” and that this translates to the neural level. Given this, along with Cohen's et al. (2012) suggestive results, research into the plasticity and educability/trainability of temperament trait effortful control could lead to methods of improving this important ability in “normals” and perhaps methods of addressing individual or group deficiencies.

Further, if people can through practice improve their ability to use reappraisal effectively, can they eventually automatize it, so that it becomes “second nature”? These questions could in fact be pursued in the case of any strategy for cognitive control. And clearly they bring the study of cognitive control into contact with work on individual differences in ability, on habit or skill acquisition, and on the shift from conscious control and practice to automatic activation [see Logan (1988) on two competing psychological models of automatization]. A related question for further investigation is whether one can improve in the use of a given strategy by mental rehearsal (i.e., via imaginative simulation of anticipated situations of decision or action) as one can with many motor skills (see Gunaratana, 2002; Lutz et al., 2009).

Regarding neural underpinnings, it would be particularly interesting to know whether the system of executive control involved in the practice or application of reappraisal was the same in different domains. Does it differ, for example, depending on whether the process to be cognitively controlled is itself psychological (as in cognitive control of affect or of unwanted thoughts), or physical (as with consciously monitored practicing in sports or music)? Comparison of research on cognitive control of affect with results in other domains would bear also on the question of whether or not there is a single, general-purpose system of executive control, and if so, how it relates to domain-specific systems of cognitive control. Indeed, accumulating neuroscience evidence indicates that executive control functions are not implemented within a unitary system but may instead reflect the operation of multiple information processing systems (Koechlin et al., 1999, 2000; Miller and Cohen, 2001; Braver et al., 2003; Sakai and Passingham, 2003; Curtis and D'Esposito, 2004; Braver and Barch, 2006; Crone et al., 2006; Dosenbach et al., 2006; Koechlin and Summerfield, 2007; Barbey et al., 2012).

This brings us to an example of contemporary research with the kind of broad scope we think necessary in the long run. Posner et al. (2008) investigate the genetic and neural basis of a human executive attention network. More specifically, their work combines recent developments in genetics, fMRI, and behavioral testing to track the development of an attentional network in early childhood. The neural systems involved coincide with results of fMRI studies of cognitive control in adults, at least insofar as it includes anterior cingulate cortex and portions of lateral prefrontal cortex; for reviews see Botvinick et al. (2001), Miller and Cohen (2001), Ramnani and Owen (2004), Barbey et al. (2009), and Barbey and Patterson (2011). The experimenters also correlated their results with parental reports of children's ability for self-regulation of cognition and emotion. One long-range goal was to investigate the role of genes and experience in the emergence of this network in 4-year-olds, and how it might constitute a general foundation for acquiring a series of more specific skills during early school years. From our point of view such a network would be an important element also in the development and exercise of any of the strategies for cognitive control surveyed so far. This would be particularly important if ways were found to systematically strengthen such a system of executive attentional control, whether in children or adults.

Finally, the case of reappraisal already suggests ways in which psychological and neuropsychological research itself can make important if indirect contributions to direct practical efforts at cognitive control in concrete circumstances. These potential contributions include (1) discovering or verifying experimentally the efficacy of a given strategy for cognitive control; (2) evaluating experimentally the pros and cons of different strategies (e.g., the potential undesirable “rebound” effect of suppression (Wegner et al., 2004); (3) exploring the effectiveness of different strategies with respect to control of different types of affect (e.g., anger management, stress reduction, curbing one's enthusiasm); (4) exploring the possible plasticity and educability of the ability to use a given strategy; (5) investigating individual and group differences with respect to capacities for control (Kanske, 2012, this issue), along with possible ways to address deficiencies [e.g., in very young or old populations; see esp. Braver (2012)]; (6) testing methods by which different strategies might be taught, learned, practiced, and even perhaps routinized so they themselves become second nature. Cognitively controlled research in all these areas can potentially discover effective means of cognitive control and explore possibilities for helping children and adults improve their ability to exercise particular strategies of control.

### Cognitive control in extended deliberation

The strategies for control surveyed so far treat conflict between reasoning and affect as a simple one-on-one encounter. But when a decision is important and an agent has the time, and the cognitive and imaginative resources to think things through, the factors involved can multiply quickly. This “extended deliberation” predictably produces a mix of reasons and intuitions/impulses/emotions on both sides of the question. (By “extended deliberation” we mean roughly a process of identifying and weighing all the significant pros and cons pertaining to a particular judgment or choice—or at least, the significant factors an agent can find by making an honest and unhurried but not clinically obsessive effort.) Extended deliberation typically includes multiple forms of interaction among those various factors, and multiple points in time at which these interactions occur. In addition, different factors and interactions may well engage different neural mechanisms if different specific forms of cognitive control come into play. Extended deliberation is more difficult to study, either behaviorally or via neuroimaging, than specific, “one-on-one” control strategies. After analyzing the process a bit further we will venture some suggestions about how it might nonetheless be approached empirically. For present purposes we will look more closely at deliberation arising from a conflict between one's “better judgment” and an affective impulse, but bear in mind that it can also arise from conflict between affective impulses, or even from conflicting reasons.

#### Reasoning recruiting affect

One frequent feature of extended deliberation is the recruitment of supporting affect by reasoning. When we try to think of the reason(s) in favor of or against some judgment or action we ordinarily think of the consequences that would or would not ensue, depending on whether or not we performed the action. These consequences are typically valenced—i.e., they include positive or negative affect. A common mechanism for the evocation of affect is the imaging or mental simulation of actions and their likely consequences (AristotleBarnes, 1984; Barsalou, 1999). Do we go to the birthday party as promised, or do we accept the free tickets to the ball game we were just unexpectedly given? Suppose we have an emotional impulse to go to the ball game (it is a “big game” against our arch-rival) and don't really want to go to the party anyway (last year's was extremely boring). On the other hand, we ought morally speaking to attend the party, not only because we said we would, but also based on the imagined responses of other attendees—their delight if we show up, their disappointment if we do not. Other emotionally valenced considerations then naturally present themselves, e.g., the shame or guilt we would feel if we broke our promise, or the guilt we would feel at disappointing the hostess, who has always treated us well. So already the option favored by moral reasoning has recruited two “rational” considerations (“a promise was made and must be honored”; “you shouldn't mistreat someone who has treated you well”), but also a variety of emotionally charged scenarios to bolster those rational factors and to counter our emotional impulse to go to the ball game. Real-life extended deliberation will usually be more complex still, since the initial gut response will also recruit both affect and reasoning to its cause.

In a nutshell, in extended deliberation both (or all) sides of a conflict will recruit both affect and “cooler” considerations. Moreover, as deliberation develops over time, opposing factors will interact: although some newly recruited factors will be simply additional considerations meant to add cumulative force to one side or the other, some will be direct responses to considerations advanced on behalf of the opposing viewpoint while still others will be replies to those responses.

#### Scanners in the fog of war

These are not mere possibilities, but facts of life when an individual, a pair of agents such as a married couple, or a group of people must think through a difficult decision, trying to identify and evaluate the pros and cons of various possible options. These cases raise an obvious problem for the use of brain imaging studies to address the question of the possibility and manner of interaction between affect and cognition. The problem is not just the perennial one of whether and how one can make a clear cut distinction between affective and cognitive processes. The further problem is that even if we are able to determine during actual deliberation that one sort of underlying neural system or structure (e.g., dorsolateral prefrontal cortex vs. amygdala) shows a greater increase in activity at a given time than the other, this will not tell us *which* particular reasons or which affects actually account for the observed increase in activity (for a review, see Dolcos et al., 2011). After all, there will be both sorts of factors on both (or all) sides. Moreover, heightened activity in either the dorsolateral prefrontal cortex or the amygdala might well reflect more than one simultaneous process of cognition or feeling.

Future research into these matters might well begin by using experimental material suitable for extended deliberation, preferably issues that people actually do deliberate and debate. Then keep a running, temporally fine-grained self-report record of conscious steps of deliberation while the underlying brain activity is being recorded. Recent work on social interaction suggests a promising variant on the usual one-participant-at-a-time setup: include two participants, each in a scanner and each able to communicate with the other via keyboard and screen as they discuss some question that is of actual concern to them (for a review of the technique and some of its technical problems, see Montague et al., 2002). Comparison of sufficiently precise neuroimaging data with real-time records of deliberation could potentially throw light on some—though probably not all—of the questions listed in the previous paragraph.

Among the many additional questions raised by extended deliberation we mention only the most fundamental of these, and note that it applies also to the simpler cases of cognitive control reviewed above under “Reactive Regulation of Affect and Emotion.” Can reasoning itself have causal or motivational force, for example in countering affect or impulse, or must it arouse/recruit affect in order to have such influence? On the surface extended deliberation appears to pit reasons against reasons, affect against affect, and reasons against affect. We tend to think of valenced affect as itself motivational. But are we to think of “rational considerations” as possessing some kind force or weight in and of themselves?

Philosophers have debated this question for centuries, but today it may at least in part be addressed empirically. Relatively simple cases of control such as reappraisal or distraction might already shed light on the issue, for in each type of cognitive control one could ask whether its conscious exercise involves arousal of affect in its own support. Neuroimaging evidence would be critical, since it could in principle reveal activity in affective systems even if an agent did not report any conscious experience of affect arising in support of the initial “cooler” side of the conflict. It would be important also to compare successful attempts at control with unsuccessful ones, to see whether they correlated systematically with greater or lesser activity or some change in patterns of activity in affective systems. As usual, however, matters quickly grow more complicated. Should consciously felt *effort* at control be counted as affect—e.g., exerting “will power” in the face of temptation, or “maintaining resolve” through a prolonged effort? If so, is the feeling of exerting effort the sort of affect that reason must rely on if it is to have causal influence on judgment or behavior? If so, how (neurally speaking) does reasoning summon this sort of exertion? On the other hand, would reasoning not in fact have to have some other source of influence in cases where we do not experience any feeling of effort? Or do we then postulate unconscious effort and look for neural underpinnings? Along the same lines, if people can through practice become better at using a particular strategy of control should we not predict that they will with increased mastery be able to exercise *greater* control with *less* effort? There are many more related questions awaiting investigation, but since this paragraph was supposed to formulate “just one,” we will leave off here, and turn to proactive strategies for control.

## Thinking ahead: proactive cognitive control

### Preventative measures

In everyday experience it is not necessary to be entirely reactive with respect to emotion. [We borrow the terminology of “reactive” and “proactive” from (Braver, 2012), but give it much broader application.] As Aristotle pointed out (AristotleBarnes, 1984), people are often aware of their own susceptibilities and weaknesses, and they can take reasonable steps to avoid situations that are likely to arouse unwanted impulses or thoughts. An alcoholic may avoid drinking parties, or take one route home rather than another to avoid passing directly by the corner tavern.

A quite different means of control is described in a very recent pilot study of the effects of mindfulness meditation on emotion and cognition (Froeliger et al., 2012, this issue). Experimental results suggest that although practitioners exhibit limbic reactivity to negative emotional stimuli, this reactivity appears not to have the usual downstream effects on later mood states. Especially interesting is the authors' finding concerning these practitioners' performance on the Stroop test following presentation of potentially interfering negative emotional images: yoga practitioners may employ “selective implementation of frontal executive-dependent strategies to reduce emotional interference during competing cognitive demands and not during emotional processing *per se*.” Further research is needed to determine whether meditation can enhance capacity for control with regard to additional sorts of contexts, tasks, and potential distractors.

### Positive affect and the goldilocks effect

Proactive measures are not always aimed at potentially deleterious affect. If we are aware that appropriate emotion would be useful in enhancing thought or action we may try to stimulate that emotion. Knowing that a blasé attitude is detrimental to cognitive performance, a student might try to “get psyched” for an exam (via self-exhortation, perhaps with caffeine supplement). Some very recent work supports experimentally the familiar notion that an appropriate level of stress can enhance performance by enhancing attentional focus or raising one's energy level (Minois, 2000). We take this case as representative of the “Goldilocks” effect, in which cognitive control aims not at suppression, distraction, etc., but at just the right level of affect—not too hot and not too cold, but just right for cognitive or motor performance. We hypothesize that this applies to other sorts of affect as well (e.g., anger, confidence) and consider the ability to tune affect by regulation up or down to be an additional area for future research. One challenge will be to identify specific strategies for modulation of affect, analogous to research on strategies for the type of cognitive control discussed in section “Reactive Regulation of Affect and Emotion” above. These tuning or modulatory strategies would presumably be different than those for simple suppression, distraction, etc., since their aim is to modify and make use of affect rather than to eliminate it from consciousness or block its effect. They would also include strategies for both raising or lowering levels of affect, where agents might well need different strategies for raising than for lowering, even with regard to the same type of affect (stress, anger, fear). A key overarching question will be *how* this modulation of affect proves helpful or the opposite: is it a matter of supporting (or interfering with) desirable levels of attention, energy, or motivation? If so, are there better, perhaps more direct or more reliable methods for achieving this? Answering these questions could have large practical implications.

### Present at the creation: inculcation (or extinction) of intuition, habits, traits, and dispositions

Not all traits or dispositions to quick responses of a given type are acquired, and not all acquired ones are acquired through the deliberate application of cooler processes of planning, training, etc. But some are, and in particular situations these deliberately acquired or shaped dispositions and habits give rise in turn to a great many fast, intuitive, or “hot” responses. Thus, slow, controlled cognitive processes that lead to underlying traits, or dispositions to a certain type of intuitive response, can have a large impact at least indirectly on particular intuitive or gut responses. We suggest that this is the single most important source of cognitive control of affect and intuition.

We sometimes consciously and on the basis of reflection decide to alter our own behavioral patterns, as with “New Year's Resolutions.” And sometimes we actually follow through. An extreme example is Benjamin Franklin's systematic program of identifying desirable and undesirable habits and personal traits, then performing actions calculated to inculcate or root them out, keeping a real-time written record of his relevant actions.

But more often the cooler processes of deciding what habits and traits we should have, as well as any thinking about how those habits are to be taught and learned, is done by others. Much of it is in fact exercised in our “upbringing” and early education in everything from correct table manners and habits of personal hygiene to virtues such as generosity, honesty, and good citizenship. The aim is to make these things second nature, so that they have a “learned naturalness” (Boyer, 1994). When that is achieved, they issue naturally, and without need for deliberation, in “intuitive” or “instinctive” judgments and actions when triggered by relevant circumstances. Here cognitive control meets developmental psychology, educational policy, “parenting,” moral development, social planning, legislation, law enforcement, and transmission of culture in general.

The processes of enculturation are numerous and closely intertwined. This is where some Social Intuitionists rightly speak of “immersion in cultural complexes” (Haidt, 2001). Still, we maintain that some of the most important aspects of these cultural complexes consist of slow, controlled cognition in various forms. To cite just a single important American example, one can point to the continuing influence of the “wisdom of the Founding Fathers” as expressed in the Constitution. That document's ideas about the basic structure of good government and certain ideals of citizenship in a democracy have been inculcated continuously ever since. In part the institutions of inculcation themselves (e.g., a system of public education) have been established along lines long ago deliberated, decided, and even built into our collective experience by deliberated legislation (e.g., laws requiring a certain level of schooling) and curriculum decisions (e.g., requiring a course in “civics”). Occasionally substantial changes have been made to the system (Amendments to the Constitution), such as extending voting rights beyond white adult males. These changes, too, have been deliberated and adopted in accordance with considered procedures set out in the Constitution itself. In addition, major issues of interpretation have arisen and been settled through deliberation and voting by the Supreme Court (e.g., rulings establishing a broad interpretation of “free speech” under the First Amendment). All these instances of deliberation and carefully weighed decision-making have had a large impact on the habits of perception, thought and action of millions of people. This is true even for those who have paid little conscious attention to these processes and would be hard pressed to explain them coherently. In the present example much of the reasoning involved occurred quite a long time ago; but its impact on our intuitions here and now in the twenty-first century is very real nonetheless. Again, although it is important to note that our latter-day judgments are often intuitive, this is only the beginning of the story, and overlooks important ways in which reasoning helps shape and control a great many of our affective responses (e.g., to voter suppression or voter fraud) and intuitive judgments (e.g., about proper political process or the scope of civil rights).

### Somatic markers and alarm bells

Finally, we consider briefly two additional proactive and long-term sources of gut responses. The first is what Antonio Damasio calls “somatic markers” (Damasio, 1996). Damasio points out that many of our decisions, judgments, and actions are at least constrained by emotionally valenced neural representations of events and associations implanted by relevant experience. Once established, they serve to circumscribe the range of viable responses or decision-making alternatives by nipping the “frame problem” in the bud. That is, cognitive or behavioral problems typically can in principle be addressed in an unlimited number of ways, whereas an agent does not have the time, resources, or need to investigate them all. Thus, the problem must be “framed” so as to limit the possibilities to a manageable field of alternatives. Agents clinically deficient in this regard tend to engage in extreme—and exasperatingly extended—processes of deliberation (Damasio, 1996). Somatic markers make normal decision-making possible by automatically closing off various alternatives without our having expressly to consider them. At the same time they can bias one option over another by automatically giving it a positive or negative valence.

These markers are sometimes distinguished from more intensely valenced automatic responses, especially aversive ones, such as disgust or moral repugnance. Greene and colleagues (Greene et al., 2001; Greene, 2007) relate the difference between somatic markers and alarm bells to the traditional philosophical distinction between deontological and utilitarian ethical judgments. Roughly put, deontological judgments are based on specific and “absolute” rules or principles; e.g., “it is impermissible to kill an innocent bystander even in order to save five lives, because murder is always wrong.” Utilitarian judgments allow specific moral rules to be overridden by considerations of the “greatest good for the greatest number”; e.g., “it is permissible to kill an innocent bystander to save five lives, because the overall good outweighs the harm.” The “Trolley Problem” has been familiar in philosophy for a few decades and has recently formed the basis of numerous psychological experiments. We put aside for present purposes the question of how much the Trolley Problem can tell us about actual moral choices. In any event there are now many variants on the problem, but the most basic version is this: You see a trolley car headed toward a group of five people standing on the tracks. If you don't do something the trolley will kill them all. However, you can throw a switch and divert the trolley onto a side track, so that it will only kill one person. Do you intervene, or just let things take their course? Most respondents say it is morally permissible to intervene in order to save the greater number. Now vary the scenario: you can save the five people only by directly pushing a large person off a bridge and onto the trolley track. Is it permissible to push him off or not? Most people say they do not think this permissible. Why is that, if in both cases it is a matter of sacrificing one person to save five? Some philosophers and psychologists suggest that the throwing-the-switch scenario activates only a cooler (i.e., somatic marker based judgment) utilitarian judgment, whereas the pushing-a-man-off-the-bridge scenario triggers a basic gut aversion (i.e., alarm bell response) to harming someone through direct personal action.

Greene and colleagues found that fMRI scans suggest a systematic neural difference in the type of affective input commonly linked to these two styles of judgment: deontological-style assessments are commonly driven by alarm bell responses, whereas utilitarian-style judgments are influenced by relatively more subtle somatic markers (Greene et al., 2001). However, from the point of view of how reasoning can influence affect/intuition/gut response, it perhaps goes without saying by now that the main issues are the extent to which somatic markers and alarm bells can be implanted deliberately under the control of executive processes, the extent to which they are open to beneficial modification by top–down executive processes, and the like. No doubt somatic markers and alarm bells are sometimes established by experience willy-nilly—as when one receives a conk on the noggin from a playground swing, or an indignant reproof from a proper young lady (e.g., a slap in the face, if that is not entirely a thing of the past). But just as with habits, somatic markers and alarm bells need not be acquired or inculcated thoughtlessly. We suggest that in fact they are often established as a result of deliberated, reasoned judgment on the part of others—for example, parents who give thought to what sort of markers ought to be established in their children, and to how these markers are to be established. Punishment and reward, admonition and encouragement, censure and praise, succeed in part by deliberately establishing appropriate somatic markers that help guide future decisions and actions. If successfully implanted, these markers in turn contribute to the shaping of many intuitive responses in concrete situations. The obvious parallel with traits or habits is not coincidental, since the selective laying down of somatic markers and subsequent repeated activation of them is part and parcel of much of our habit formation.

The same holds for “alarm bells,” even though they may seem more deeply visceral, inflexible, and perhaps more closely tied to evolved genetic origins. The example of disgust is a good case in point. It is one of our loudest alarm bells, and one closely allied with certain moral responses (see, for example, Nichols, 2004). Some of our disgust reactions (e.g., to the smell of putrefying flesh) are evolved, and are extremely resistant to any process of extinction. But some, including reactions of disgust, fear, etc., are just as clearly cultural, as with Mary Wollstonecraft's moral disgust at the sight of “a fine lady clasping a lap dog to her bosom” (Wollstonecraft, 1792). Behind this intuitive alarm bell of moral disgust lies a good deal of experience, and reflection on the moral fatuousness (or worse) of “fine” ladies. Such reflection can help change the way we conceive a type of person or situation, so as to trigger an alarm bell that it did not trigger in the past. There was a time, perhaps, when we admired the fine lady, and intuitively found it endearing that she clasped her little dog to her bosom.

The neuroscience evidence just mentioned (Greene et al., 2001; Greene, 2007) does, however, raise one new and important issue. With regard to types or styles of moral judgment in particular we would caution against thinking of deontological and utilitarian approaches as exhaustive alternatives. Moral traits and habits, including those we label “virtues” and “vices,” constitute a very important source of moral perceptions, judgments and actions, and they do not fit easily into a simple deontic/utilitarian scheme. Moral philosophy has in recent decades seen a resurgence of interest in “virtue ethics” as an alternative to deontic and utilitarian theories of how one is to live a morally good life. The approach goes back to Plato, Aristotle, Aquinas, et al. A pivotal modern work is Mac Intyre (1984) but there is now a voluminous literature on the topic. In virtue ethics, virtues and vices are construed along the general lines of habits or character traits. Consequently we have strong reservations about framing psychological or neurological research—or interpreting the data from such research—in terms of a deontological vs. utilitarian dichotomy.

As for the use of scanning evidence to support such a dichotomy, we suggest that although the Trolley Problem may in fact pose a fairly clear conflict between deontological and utilitarian principles, other sorts of moral judgment do not. Moreover, in many circumstances the same moral conclusion would be consistent with both deontological and utilitarian principles. For present purposes, however, let it suffice to say that future research should recognize the option for interpretation afforded by virtue ethics (and still others would be possible), and consider carefully how differing responses to given types of experimental materials might correlate with different underlying bases of moral judgments. Certainly recognizing the importance of moral traits and habits opens the door to a great deal of further research, and to the forging of connections with already established research on learning, practice, and automatization of moral and other responses.

## Conclusion

Our point of departure for a broad consideration of cognitive control was a particular interpretation of moral judgment as virtually always intuitive. We conclude that although there is much truth in this description of such judgments considered in their immediate context, it is necessary to look beyond that context when addressing the question of whether and how reasoning might potentially influence moral and other intuitions, or whether rational control in this area is an illusion (Haidt, 2001). A broader perspective reveals numerous ways in which reasoning can and very often does influence affective response and intuitive judgment, even if that influence is in many cases indirect. Much of our discussion, especially that of measures for proactive control, constitutes a response to the question, “Where do our intuitive judgments come from?” Putting aside other factors, we have focused for present purposes on ways in which reasoning, planning, cognitive monitoring, and the like play a substantial role in establishing the underlying sources of intuitive judgments. Chief among these sources are the diverse habits, traits, skills, expertise, and dispositions that give rise to fast, automatic, sometimes “hot” responses in particular circumstances. Of course these sources are not inculcated entirely through slow, cognitively controlled processes. But many are acquired or shaped to a significant extent in that way, and this constitutes a larger field for the control of the fast and hot by the slow and cool than do the reactive strategies that have up to now received more attention in the literature on cognitive control.

In addition there is a great deal of work to be done on related issues regarding cognitive control: strategies for up-regulation of affect as well as for suppression, reappraisal, etc.; modulation or tuning of affect to enhance cognition or action; strategies for improving or automatizing abilities to apply strategies for modulation or control; and others. These all bear on situations of moral judgment, but they in fact apply to perception, judgment and action of all sorts and call for further investigation as important aspects of the wide and complex domain of cognitive control.

### Conflict of interest statement

The authors declare that the research was conducted in the absence of any commercial or financial relationships that could be construed as a potential conflict of interest.
